# Computer classes and games in virtual reality environment to reduce loneliness among students of an elderly reference center: Study protocol for a randomised cross-over design

**DOI:** 10.1097/MD.0000000000005954

**Published:** 2017-03-10

**Authors:** Thaiany Pedrozo Campos Antunes, Acary Souza Bulle de Oliveira, Tania Brusque Crocetta, Jennifer Yohanna Ferreira de Lima Antão, Renata Thais de Almeida Barbosa, Regiani Guarnieri, Thais Massetti, Carlos Bandeira de Mello Monteiro, Luiz Carlos de Abreu

**Affiliations:** a Laboratório de Delineamento de Estudos e Escrita Científica da Faculdade de Medicina do ABC, Santo André; b Neurologia Clínica, Setor de Doenças Neuromusculares, Universidade Federal de São Paulo; c Departamento de Pós-graduação em Ciências da Reabilitação; d Faculdade de Saúde Pública. Universidade de São Paulo, Sao Paulo, Brazil.

**Keywords:** augmentative and alternative communication, elderly, information and communication technologies, serious games, social inclusion

## Abstract

**Introduction:**

Physical and mental changes associated with aging commonly lead to a decrease in communication capacity, reducing social interactions and increasing loneliness. Computer classes for older adults make significant contributions to social and cognitive aspects of aging. Games in a virtual reality (VR) environment stimulate the practice of communicative and cognitive skills and might also bring benefits to older adults. Furthermore, it might help to initiate their contact to the modern technology. The purpose of this study protocol is to evaluate the effects of practicing VR games during computer classes on the level of loneliness of students of an elderly reference center.

**Methods and Analysis:**

This study will be a prospective longitudinal study with a randomised cross-over design, with subjects aged 50 years and older, of both genders, spontaneously enrolled in computer classes for beginners. Data collection will be done in 3 moments: moment 0 (T0) – at baseline; moment 1 (T1) – after 8 typical computer classes; and moment 2 (T2) – after 8 computer classes which include 15 minutes for practicing games in VR environment. A characterization questionnaire, the short version of the Short Social and Emotional Loneliness Scale for Adults (SELSA-S) and 3 games with VR (Random, MoviLetrando, and Reaction Time) will be used. For the intervention phase 4 other games will be used: Coincident Timing, Motor Skill Analyser, Labyrinth, and Fitts. The statistical analysis will compare the evolution in loneliness perception, performance, and reaction time during the practice of the games between the 3 moments of data collection. Performance and reaction time during the practice of the games will also be correlated to the loneliness perception.

**Ethics and Dissemination:**

The protocol is approved by the host institution's ethics committee under the number 52305215.3.0000.0082. Results will be disseminated via peer-reviewed journal articles and conferences. This clinical trial is registered at ClinicalTrials.gov identifier: NCT02798081.

## 1 Introduction

Individual aging, or senescence, is a progressive process that affects all systems structurally and functionally, compromising speed, accuracy, strength, stability, and coordination.^[1]^ These difficulties suggest differences between older and young adults regarding the ability to process information and learn.^[2]^ This can happen in both motor and cognitive tasks, leading to a decrease in the communication capacity of older adults, consequently reducing social interactions, and increasing loneliness.

Loneliness can be defined as perceived social isolation: the feeling of missing intimate interpersonal relationships (but not necessarily an actual lack of social interaction).^[3]^ With ageing, people become particularly vulnerable to loneliness due to deteriorating physical health, the death of spouses and partners, being more likely to live alone, and having fewer confiding relationships.^[4]^ Therefore, with ageing, people should learn how to use communication tools, which are new or already known, in different ways as an attempt to overcome loneliness.

Information and communication technologies (ICTs) are important instruments to access contemporary culture and to feel socially included. With the advent of mobile technology, the definition of communication and the means of social interaction are changing rapidly. Therefore, ICT can be an important tool to reduce loneliness among older adults.^[5]^

In general, the younger generation has confidence in using technology and assimilates the changes easily as, since their early years, they have explored electronic toys and/or played with mobile phones. However, the older generation, born before the development of the digital world, has more difficult in accessing these technologies.^[6]^ In addition, some elderly individuals may lack the motor skills to easily use digital devices.

Therefore, interventions to make ICT more accessible for older adults must focus on helping them acquire knowledge needed to maximize motor performance and social interaction, as both cognitive and motor skill are required to readily use ICT devices. Although they experience functional loss, older adults have the capacity to acquire new abilities and their performance can be similar to young adults.^[7]^

Research on computer classes to elderly, shows that, given appropriate structures of teaching and learning specific to older adults, elderly individuals can learn these skills even though aging can make it more challenging.^[6]^ The psychosocial impact on older adults who learned how to use the Internet was positive, showing a trend toward decreased loneliness and depression.^[8]^ One possibility for helping older adults learn effective use of ICT is via virtual reality (VR), which in general is a useful tool to study, evaluate, and rehabilitate cognitive processes and functional activities.^[9]^

VR allows the performance of new tasks and might stimulate the practice of communicative and cognitive skills, benefiting functional improvement and initiating the contact of older adults with the modern technology. Still, game software can stimulate and, also, store responses, differently from other preconized methods.^[10]^ Virtual games and virtual environments can be used as physical, cognitive, or psychological interventions to enable function and fun for different people.^[11]^ By using VR, it is possible to promote flexibility and control in task administration, increasing the chance of transferring an acquired ability in an efficient and safe way.^[12]^

Regarding VR utilization for older adults, there are studies on postural control,^[13]^ balance, fall prevention, physical activity incentives, and functional performance.^[14]^ The positive effects of using a VR game included improving physical function, decreasing depression, and increasing cognition and quality of life in older adults. Improved socialization and motivation to exercise were also reported.^[15]^ Most of the studies that assess older adults in virtual tasks were done with older adults with acquired physical and cognitive diseases, and the healthy older adults were only part of the control group.^[16]^ However, the use of VR as a means of facilitating the use of computers and reducing loneliness of healthy older adults is scarce.

Considering the settings presented, we believe that motor and cognitive ability acquired through VR games might facilitate computer utilization, which is a valuable communication tool.^[17]^ Therefore, we intend to assess, through a loneliness scale, a group of older adults that are initiating computer classes for beginners. The effects of practicing games using VR within computer classes will be compared to the impact of conventional computer classes. Games with VR, requiring a variety of different motor and cognitive abilities will be used to identify whether evolution in loneliness, is related to the practice of tasks using virtual ICTs. We hypothesized that the practice of VR games added to conventional computer classes will lead to an improvement in motor ability and less loneliness of older adults.

Thus, the main objective is to evaluate the effects of practicing games in a VR environment during computer classes on loneliness among students of an elderly reference center (ERC). Moreover, in students initiating computer classes for seniors, we aim to: evaluate the evolution in time of response during the practice of VR games; evaluate the evolution in performance of practice of VR games; and evaluate the relation between motor ability to loneliness.

## 2 Method

This study protocol followed the Stardard Protocol Items for Randomized Trials (SPIRIT).^[18]^ This study will be a prospective longitudinal study with a randomized cross-over design which will use games in a VR environment within computer classes to reduce loneliness among students at an ERC.

### 2.1 Study population

It will be a convenience sample. The participants will be students in beginning level computer classes for seniors in the CRI in Ribeirão Pires, Sao Paulo, Brazil.

Because no study was found using the short version of the Social and Emotional Loneliness Scale for Adults (SELSA-S) before and after any intervention, sample calculation was not possible. However, there will be a minimum of 23 participants in this study, based on the largest sample that used VR in older adults found in the literature.^[13]^

The CRI Ribeirão Pires offers cultural, sportive, and intellectual activities for people over 50 years old, as used in the multicenter Studies on Global Ageing and Adult Health project of the WHO.^[19]^

### 2.2 Inclusion and exclusion criteria

Initially, all students from beginning level computer classes for seniors at the CRI in Ribeirão Pires will be invited to participate.

Those who attend less than 75% of the classes between the assessments and/or do not complete the 3 moments of assessment will be excluded from the research. Individuals with motor impairment of the upper limbs which prevents them from practicing the games or a score lower than 23 in the mini-mental exam^[20,21]^ will also be excluded.

Any CRI student who is absent from class 3 times without a plausible excuse is excluded from the course and, therefore, will be excluded from the study. This helps ensure intervention adherence.

To reduce the likelihood that any student will feel harmed, the same intervention will be offered to all the participants. Those who match the exclusion criteria, but are willing to participate, will be excluded during data analysis.

### 2.3 Expected risks

The intervention proposed here generates minimal risks, as it involves noninvasive measurements. The informatics classes, as well as game practicing, will be done in the usual classroom. If the participant feels any discomfort, the proposed tasks must be stopped and the participant will be aided by the responsible professionals. Data will be collected in a private room, in the same place the participants have the informatics classes, attended only by the researchers and professionals skilled to help during its execution.

Although the procedure is noninvasive and the risks are minimal, the possible risks to physical or mental health are: eye strain due to sustained computer use, imbalance or pain during the movements imposed by the virtual games, or feeling sick or tired. Some embarrassment for not being able to or not understanding how to play the games may also happen. In any of these cases, the participant may withdraw from participation.

### 2.4 Expected benefits

It is expected that repeated use of VR games will influence the motivation of the participants to use computers and allow them opportunities to acquire or develop motor abilities needed for it in an enjoyable way and, consequently, loneliness will be reduced.

Participation in the research will also contribute to identify how practicing different virtual games can influence performance of computer tasks and achieving the goal of every game. This may allow health and education professionals to adjust their practices in accordance to the characteristic of their attended population. If the addition of games with VR in the computer classes has positive results, they may be introduced as a standard practice in computer classes, for example.

### 2.5 Intervention

There are 6 groups of computer classes for beginners offered by CRI, with a maximum of 10 students each. The classes last 2 hours, twice a week and include basic computer operations, the use of Word programs and introduction to the Internet.

For the intervention, the 6 classes of computer lessons for beginners will be randomly assigned into 2 groups, group 1 (G1) and group 2 (G2), with the same number of classes for each group.

**Figure 1:**
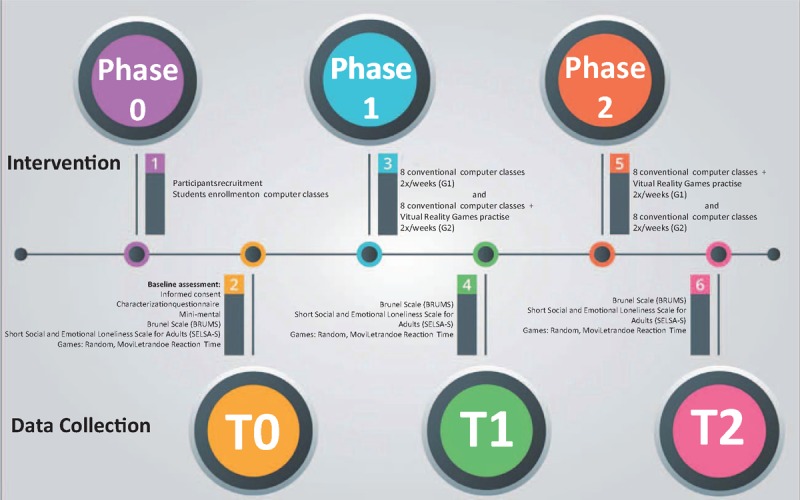
Study flow.

The intervention will be divided into 2 phases, as shown in Fig. 1. In phase 1, G1 will have 8 conventional computer classes, while G2 will have 8 computer classes which will include 15 minutes of practicing games in a VR environment, using a physical interface (keyboard or mouse). In phase 2, intervention will be crossed between the groups. Therefore, G2 will have 8 conventional computer classes, while G1 will have 8 computer classes which will include 15 minutes of computer game practice. The games used for intervention (Labyrinth, Fitts, Motor Skill Analyzer [MOSKA], and Timing Coincident) will be described in the section Games for training.

### 2.6 Data collection

Data collection will be in the usual computer classroom and done by the researchers and expert professionals available to help during its execution. The participants will be seated comfortably in an adjustable chair, or in their own wheelchair, whichever is the case. In order to protect confidentiality during and after the trial, the participants will be assigned numbers during data collection and their names would not be used during data analysis.

Data collection will be done at 3 times: moment 0 (T0) – before the beginning of the computer classes; moment 1 (T1) – after Phase 1; and moment 2 (T2) – after phase 2. The study flow is on Fig. 1.

In the first meeting (T0), the informed consent (IC) form will be presented and explained. After signing the IC, a characterization questionnaire, the SELSA-S,^[22]^ the Mini Mental,^[23]^ and the Brunel Mood Scale^[24]^ will be filled in, always with help of one of the researchers. Then, instructions for playing the VR games will be given verbally by the researcher. Participants will then play 3 games about which data will be collected: Random, MoviLetrando, and Reaction Time. The time for data collection will be up to 40 minutes.

During T1 and T2, the procedure will be the same as in T0, except for the IC explanation and Mini-mental testing.

### 2.7 Instruments

Instruments for assessment

A characterization questionnaire with information on gender, age, education, marital status, and use of computer and mobile phone will be used to characterize and homogenize the sample. Scores on the Mini-Mental Status Exam,^[20,21]^ which quickly screens and evaluates cognitive function, will be used as an exclusion criterion.

Evaluating the mood of participants may aid in evaluating their performance during the games. This hypothesis is based on findings of a negative correlation between mood and the sensory and motor functioning of subjects, suggesting that low mood might interfere with functional balance.^[25]^ Therefore, the Brunel Mood Scale was administered.

To evaluate loneliness, the Portuguese version of the short Social and Emotional Loneliness Scale for Adults, SELSA-S,^[22]^ validated for the elderly population, was used.

The questionnaires will be, preferably, filled out in an electronic format developed through Epi Info, which allows building questionnaires in a quick way and was developed by the Center of Diseases Control.^[26]^ Filling the forms directly on the computer allows defining answers that are mandatory, avoiding questions without answers. The answers will be exported to Microsoft Excel, avoiding typos.

The games used in the study, Random, MoviLetrando, and Reaction Time, will be used to assess performance through reaction time, hits, and misses.

## 3 Random

**Figure 2:**
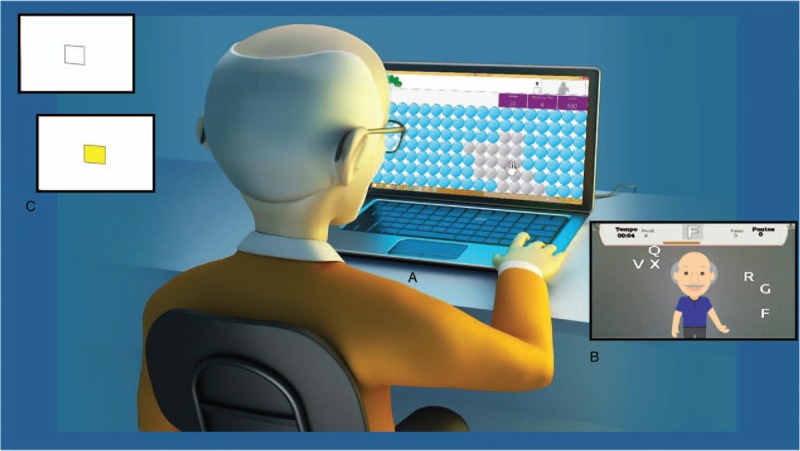
Games for assessment. (A) Random; (B) MoviLetrando; and (C) Reaction Time.

The task in Random consists of a screen that presents a set of 126 3D bubbles (Fig. 2A) arranged in rows and columns. The goal is to reach, with the mouse cursor, the bubble that changes color (from gray to red) randomly. In this case, the target is a virtual object and the interaction device to hit the target is the mouse. The game saves information on performance such as score of the targets hit and time.

## 4 Moviletrando

The game MoviLetrando^[27]^ was developed by a multidisciplinary development team at the Laboratory for Research on Visual Applications, Santa Catarina State University (LARVA-UDESC). MoviLetrando uses the concept of Projection VR, a game with movement and without an interaction device to facilitate usability.

To play MoviLetrando, a webcam and a conventional laptop are necessary. The image captured by the camera is the player himself/herself and the game scenario (Fig. 2B), helping to develop proprioception.

A reference letter or number and is generated in the upper part of the picture and, at the same time, there is a sound representing this reference symbol. A set of symbols is shown below, one of which is equal to the reference. The player's goal is to use the movement of their arms and hands to “touch virtually” the correct symbol. Between the reference symbol and the set of symbols arranged in the scene, there is a bar representing the exposure time of symbols, that is, the time that the player has to capture a symbol.

The score in MoviLetrando is given through the symbols’ arrangement and exposure time. The game works according to the concept of levels that evolve according to the player's performance. The symbols are formed with numbers, vowels, consonants, and numerical sets, which can be set individually or in combination. In addition, one can set the symbol size to be presented. For this experiment, we opted for the level that uses numbers from 1 to 10.

The player must put together strategies to achieve the correct symbol and can do this with any part of the body, usually making use of the hands.

At the end of each game, information about performance, such as hits, errors, and score are saved. It is also possible to register several people in the database. With all these data, it is possible to make an analysis of the evolution of each participant's performance during a given period of playing the game.

## 5 Reaction time

Reaction Time is a game which the participant must look to a white computer screen with a white square. Suddenly, the square turns yellow and, when that happens, the participant must press the space bar so the square turns white again. It is repeated for 14 times. The program calculates how long the participant takes to react after the square turns yellow (Fig. 2C).

## 6 Games for training

### 6.1 Labyrinth

**Figure 3:**
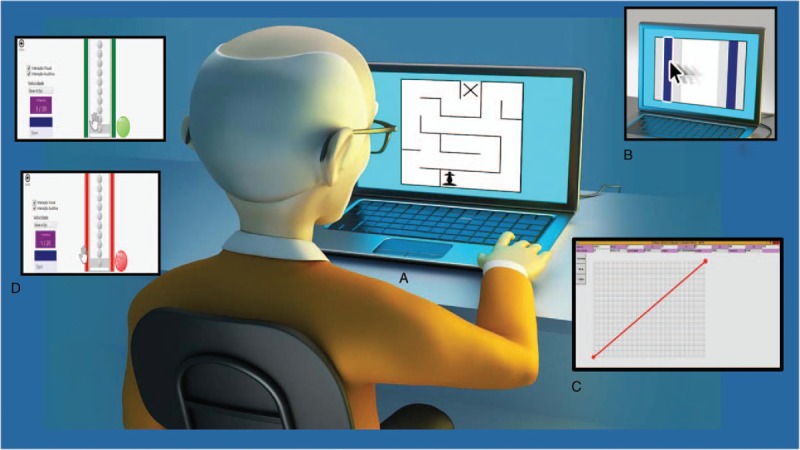
Games for training. (A) Labyrinth; (B) Fitts; (C) Motor Skill Analyzer (MOSKA); and (D) Coincident Timing.

The Labyrinth game used for training in this study was developed by the Department of Mathematics at the Federal University of Rio Grande do Sul.^[28]^ The task consists of negotiating a labyrinth with only one correct path to the exit, using the 4 arrows on keyboard. It produces a realistic labyrinth escape scenario; and therefore, the player is allowed to see only the part of the labyrinth he is in. However, to make the game easier, it is possible to opt to view the entire labyrinth during the game. The exit is indicated by an “X” (Fig. 3A). It is also possible to configure the size of the labyrinth. For this study an intermediate size of 9 × 9 was chosen.

### 6.2 Fitts

The 2nd training game was the “Fitts Reciprocal Aiming Task v.1.0 (Horizontal),” available in the public domain (http://okazaki.webs.com – available on the internet 25/04/2016) developed by Victor Hugo Alves Okazaki.^[29]^ This software represents the task proposed by Fitts law in a virtual environment (computer). For this research, the task is to use the mouse cursor of the computer to click on 2 bars, which are arranged vertically in parallel (Fig. 3B), intermittently, with the greatest possible speed and accuracy for a period of 10 seconds.

The task in this game has 3 difficulty index (DI) values. Each successive DI level is harder than preceding one, with larger spaces between the bars and/or a decrease in the size of the bar. However, due to the design of the program, only the default DI shown when the game is opened will be played during the training: that is the thinnest bar in the closer distance between them.

### 6.3 Motor Skill Analyzer (MOSKA)

The software MOSKA, here used as a game, was developed to help the diagnosis and follow-up of problems in fine motor control of the upper limbs.^[30]^

The task allows the participant to draw a straight line by clicking with the mouse on a starting point (indicated by a red circle) and moving the mouse through the line to the end point (also indicated by a red circle) (Fig. 3C). Afterwards, the software calculates the errors, distance covered, and time in milliseconds.

The drawing of the line can be defined through x and y coordinates for the start and end points. This way, the game can be set up to require the drawing of horizontal, vertical, or diagonal lines.

### 6.4 Coincident timing

Coincident timing, or coincidence of time and anticipation, refers to the cognitive ability to time the movement of a virtual object so as to arrive at a target coincident with the arrival of an object moving toward the same target.^[31]^ It is a widely used task in the motor learning area to assess the participant's cognitive development and maturation of visual and motor structures.^[32]^

It was decided to use the game software that consists of a task of virtual “coincidental timing.” The participant can receive immediate feedback of a hit or task error via different sounds, previously demonstrated to him. That game will be played during the intervention phase, using an interface with physical contact. In this case, the subject is represented as a virtual object that can be moved toward the target and is controlled via the keyboard (Fig. 3D).

The game “Coincident Timing” shows the sequence and firing speed of the cubes and records the total time of task execution (time spent to touch the sensor), as well as the time discrepancy between the arrival of the stimulus object and when the subject's virtual object arrived at the target.

## 7 Material

Evaluation games will be played on laptops with Intel Core i7-4810MQ CPU 2.80 GHz processors and 8 Gb of RAM, with Windows 8 Professional de 64 bits operating systems.

For training games, the same desktop computers that are already used by the institution for computer classes will be used.

## 8 Measures

All the data will be recorded at baseline, after 8 computer classes and after the other 8 computer classes which will include the practice of the games.

### 8.1 Primary outcomes

Data on loneliness will be collected by administering the SELSA-S and evaluated as a total and also divided in 3 subscales: romantic, social, and familiar. Higher scores indicate a higher level of loneliness, while lower scores indicate a lower level of loneliness.

### 8.2 Secondary outcomes

Data on motor ability as response time and performance in the games in a VR environment will be collected during the practice of the Reaction Time, Random, and MoviLetrando games. Lower response time and higher performance (demonstrated by higher scores and fewer errors) will indicate an improvement in motor ability.

### 8.3 Data analysis

Excel and SPSS version 2.1 for Windows will be used for data preparation and analysis. The significance level will be *P* ≤ 0.05.

The data will be presented as means and standard deviations and in frequency tables. Before the data analysis, the normality of all variables will be evaluated for skewness and kurtosis. To compare participants’ mood and loneliness, paired *t* tests, Kruskal–Wallis tests, Mann–Whitney *U* tests, or Pearson correlation coefficients will be used. The analysis will be blinded and will compare the 3 moments of data collection and the 2 groups (G1 and G2). To guard against the possibility of type 1 errors from making too many post hoc comparisons, we will carry out only those comparisons that have been planned based on our hypotheses.

## 9 Ethics and dissemination

This research project was approved by the Ethical Committee of the Faculdade de Medicina do ABC, Sao Paulo, Brazil, under the number 52305215.3.0000.0082.

This clinical trial register was titled “Virtual Reality Games for Elderly Socialization,” on August 17, 2016 at “ClinicalTrials.gov” (NCT02798081).

Signatures of all participants on the informed consent form will be obtained before data collection starts.

All the researchers will have access to the final trial dataset. The results will be available for the participants and their institution, and they will also be published in scientific journal, always keeping the confidentiality of the participants.

## 10 Discussion

Aging is a natural process of physiological, anatomical, and emotional changes that may reduce socialization and lead to loneliness. Therefore, older adults should learn how to use communication tools in different ways, aiming to maximize social interaction.

VR offers opportunities for older adults to experience various situations in an individual and safe way.^[33]^ Virtual environments also allow people with some difficulty, when connected to or immersed in them, to improve their levels of interaction with the environment.^[34]^

This study will determine whether the practice of games in a VR environment in addition to the usual computer classes reduces loneliness among students at an ERC. Changes in loneliness, reaction time, and performance in the games, as well as the relation between motor ability and loneliness, will also be investigated.
