# Computer classes and games in virtual reality environment to reduce loneliness among students of an elderly reference center: Study protocol for a randomised cross-over design: Erratum

**DOI:** 10.1097/MD.0000000000006991

**Published:** 2017-05-19

In the article, “Computer classes and games in virtual reality environment to reduce loneliness among students of an elderly reference center: Study protocol for a randomised cross-over design”,^[1]^ which appeared in Volume 96, Issue 10 of *Medicine*, the grant number appeared incorrectly in the Acknowledgements section as “2017/03374-0” and should have appeared as “2015/19922-0.”
